# Polymorphisms of CSF1 and TM7SF4 genes in a case of mild juvenile Paget’s disease found using next-generation sequencing

**DOI:** 10.3325/cmj.2015.56.145

**Published:** 2015-04

**Authors:** Judit Donáth, Gábor Speer, János P. Kósa, Kristóf Árvai, Bernadett Balla, Péter Juhász, Péter Lakatos, Gyula Poór

**Affiliations:** 1 National Institute of Rheumatology and Physiotherapy, Budapest, Hungary; 2 Policlinic of Hospitaller Brothers of St. John of God, Budapest, Hungary; 3 PentaCore Laboratory, Budapest, Hungary; 4 1st Department of Internal Medicine, Semmelweis University, Budapest, Hungary

## Abstract

Juvenile Paget’s disease (JPD) is a rare autosomal-recessive condition. It is diagnosed in young children and characterized by a generalized increase in bone turnover, bone pain, and skeletal deformity. Our patient was diagnosed after a pathological fracture when she was 11 years old. When we first examined her at the age of 30 she had bone pain and deformity in both the femur and tibia. Serum alkaline phosphatase (ALP) level, radiology, bone scintigraphy, and densitometry were monitored. Next generation sequencing (NGS) technology, namely semiconductor sequencing, was used to determine the genetic background of JPD. Seven target genes and regions were selected and analyzed after literature review (TM7SF4, SQSTM1, TNFRSF11A, TNFRSF11B, OPTN, CSF1, VCP). No clear pathogenic mutation was found, but we detected missense polymorphisms in CSF1 and TM7SF4 genes. After treatment with zoledronic acid, infusion bone pain and ALP level decreased. We can conclude that intravenous zoledronic acid therapy is effective and safe for suppressing bone turnover and improving symptoms in JPD, but the long-term effects on clinical outcomes are unclear. Our findings also suggest that NGS may help explore the pathogenesis and aid the diagnosis of JPD.

Juvenile Paget’s disease (JPD) is a rare genetic bone disease, with approximately 60 cases reported in total worldwide (1). In JPD, the function of the key proteins regulating osteoclast differentiation or function is affected. but like in Paget’s disease of bone (PDB), the exact pathomechanism is not understood. The genetic predisposition influences the function of bone cells. Though the gene candidates of the disease have not been identified yet, it is transmitted as an autosomal recessive trait (2,3). In most cases, there is a deficiency of the protein osteoprotegerin, leading to the clinical manifestations of the disease (3). No other affected genes have been identified yet.

Some patients are asymptomatic, whereas others develop complications (4). JPD manifests in infancy or childhood, characterized by greatly accelerated and disorganized bone turnover (typically at focal areas), manifesting in bone deformities, pain secondary to fractures, osteopenia of the long bones, corticomedullary indistinctness, coarsening of the trabecular bone, growth retardation, deafness due to cochlear involvement, and nerve compression syndromes (2-4). Although clinically JPD has some similarities to PDB, the early age at onset and marked bone deformities can result in different pathomechanisms.

We present a rare case of mild form of JPD with a genetic analysis – using a next generation sequencing technique – of seven target genes and regions of PDB. With the appearance of next-generation sequencing (NGS) machines, molecular biology reached a new revolutionary phase. This new technology combines high performance with much less expensive operation costs. Furthermore, we designed a novel approach to genetic testing in which we simultaneously sequenced the whole coding regions of the affected genes at the same time. Our approach was based on using an IonTorrent PGM from Life Technologies (Carlsbad, CA, USA). This benchtop sequencer belongs to the semiconductor sequencer family. It acquires the DNA sequence by detecting electric impulses created by the release of H^+^ -ions in its microchip. The solution that contains the H^+^-ions serves as a gate electrode of a transistor, a so called ion sensitive field electricity transistor (ISFET). This signal combined with cyclic addition of dNTP-s can be processed by the sequenator’s software as the incorporation of a nucleic acid, and several cycles will provide the sequence of the DNA in question. It is possible to barcode the samples with a sequence by which the software can differentiate between them. This is a great tool to sequence several samples at the same time, reducing the sequencing costs.

We used the IonTorrent PGM (Life Technologies) to examine all genes known to play a role in the development of the different subtypes of Paget’s disease. Also, we treated our patient with zoledronate therapy, which makes this the second study to describe administration of such a medication in juvenile Paget’s disease (1).

## Patient, materials, and methods

### Case report

We present a case of a 30-year-old woman with JPD. Her parents, grandparents, and two siblings had no history of any bone disease. They had normal alkaline phosphatase (ALP) level and had no known clinical bone abnormalities. However, we were unable to submit them to genetic testing. When the patient was 11 years old, she had a non-traumatic fracture on the left tibia. At that time, the serum ALP activity was elevated to 414 U/L. The bowing deformities of her lower extremities were noted and the diagnosis of JPD was made. Her bone age was normal. She received calcitonin therapy for six months and according her mother she tolerated the injections without apparent side effects and seemed to have less pain in her lower limbs. After this, she had no clinical progression for nineteen years.

**Figure 1:**
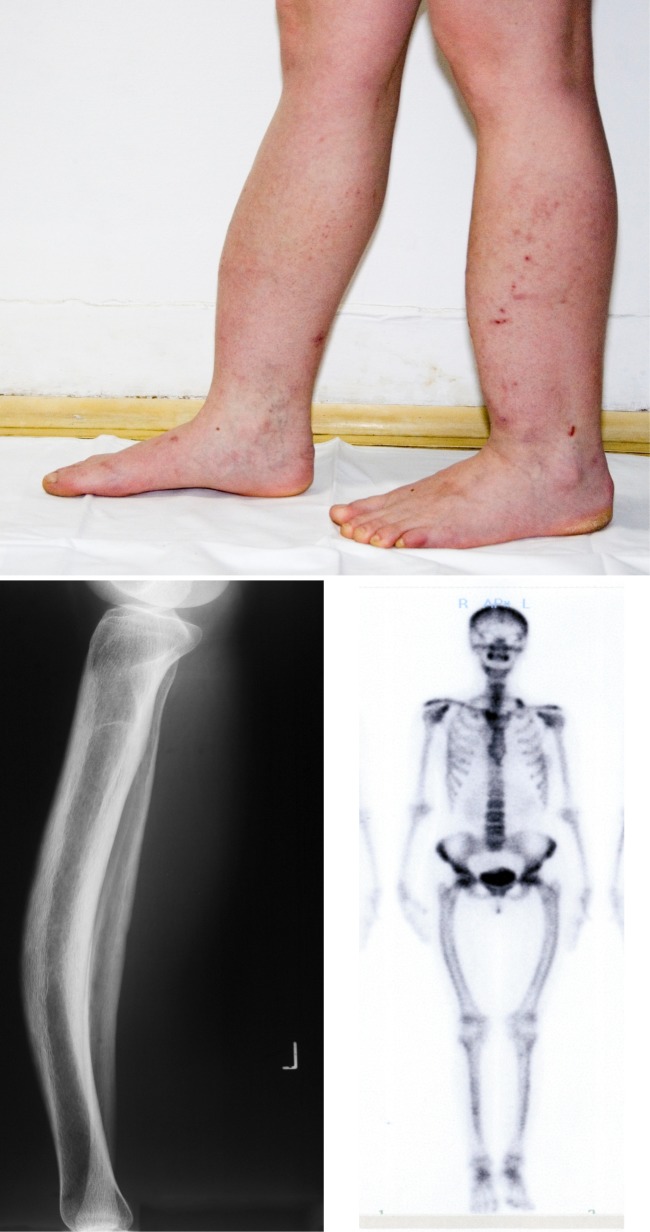
Counterclockwise from the top: Photograph showing deformity of the left and right tibia. Radiograph of the left tibia showing osteosclerosis and osteolysis. Radionuclide bone scan showing increased tracer uptake in both femur and tibia.

In May 2011, she was referred to the National Institute of Rheumatology and Physiotherapy suffering from fatigue and bone pain in her lower extremities. Physical examination showed normal vital signs, weight of 47 kg, and height of 156 cm. She had marked scoliosis of the lumbar spine and the lower limbs showed anterior bowing of both femora and tibias (Figure 1). She had no difficulties in hearing and no eye or neurological problems. The study was approved by the National Institute of Rheumatology and Physiotherapy Committee of Research Ethics, and the patient gave written informed consent.

### Mutation analysis – Ion Torrent sequencing

Genomic DNA was isolated from 200 µL of peripheral blood using Reliaprep Blood gDNA Miniprep System (Promega, Fitchburg, WI, USA). Target genes and regions (DCSTAMP, SQSTM1, TNFRSF11A, TNFRSF11B, OPTN, CSF1, VCP) (Table 1) were selected after carefully reviewing the literature (5-8) and the information in NIH Genetic Home Reference site (*http://ghr.nlm.nih.gov*/).

**Table 1:** Target genomic regions and coverages

Target identifiers	Target length (bp)	Missed by the assay designer algorithm (bp)	Coverage (%)
TM7SF4*	1413	0	100
SQSTM1	1372	22	98.4
TNFRSF11A	1851	192	89.63^†^
TNFRSF11B	1206	0	100
OPTN	1734	57	96.71
CSF1	2342	61	97.4
VCP	2421	46	98.1
rs9533156	100	0	100
rs9525641	100	0	100
rs3742257	100	0	100
rs3102735	100	0	100

*also known as DCSTAMP.

†the uncovered region of TNFRSF11A was analyzed by conventional polymerase chain reaction and agarose gel electrophoresis in order to find known deletions/duplications.

Amplicons were designed using the AmpliSeq Designer 1.2 software (Life Technologies), targeting the complete coding sequence of each gene of interest (design ID: IAD27911). Amplicon library was prepared using Ion AmpliSeq Library Kit 2.0 (Life Technologies). The emulsion polymerase chain reaction (PCR) with Ion Sphere Particles (ISPs) was run using automated template preparation system (Ion One Touch, Life Technologies). Non-templated beads were removed in a semi-automated enrichment step using Ion One Touch ES instrument (Life Technologies). ISPs were loaded into Ion 314 chip and the sequencing runs were performed with 260 flows on Ion Torrent Personal Genome Machine (PGM).

Data from the Ion Torrent run were analyzed using the platform specific pipeline software, Torrent Suite v3.2.1 (Life Technologies) to base-calling, trim adapter and primer sequences, and to filter out poor quality reads. The variants were reviewed and annotated using dbSNP *(http://www.ncbi.nlm.nih.gov/projects/SNP/*) database. Missense variants were validated by Sanger sequencing. The Sanger sequences data were investigated using ABI Sequence Scanner 1.0 (Life Technologies). The amplification primers were the following: rs1058885-F: TCCCAGAAGAAGCCTCTGGA, rs1058885-R: GCAGGTGGAAGACAGACTCC, rs3802204-F: actgagtacaaaaatggcatgca, rs3802204-R: acaccaccatcttggcctta.

The sequencing run achieved 196 777 reads, generating 18.1Mb of sequencing data. The average base coverage depth was 1037 and the 1-fold target coverage was 97.11%, and the 100-fold coverage was 91.83% with a mean raw accuracy of 99%. The uniformity of coverage was 87.52%

## Results

### Laboratory, radiographic, and densitometry findings

Six months before the patient was referred to the hospital, she had rapid bone turnover with elevated ALP levels of 570 U/L (reference range: 98-280 U/L), which after the bisphosphonate treatment declined to 334 U/L. The serum level of osteocalcin was 72 pg/mL (reference range: 11-43 pg/mL) and after treatment it declined to 25 pg/mL. Beta-crosslaps were elevated to 1347 pg/mL (reference range: 10-594) and declined to 731 pg/mL. At the first examination in our center, vitamin D deficiency was diagnosed. The 25 OH vitamin D3 level was decreased to 7 ng/mL (reference range: 23-60 ng/mL), and after vitamin D supplementation it increased to 57 ng/mL. Serum calcium and phosphate levels were within the reference range.

The x-ray tests showed involvement of both lower extremities – bowed long bones with cortical thinning and hypomineralization of the trabecular bone. Bone scan revealed increased uptake in the right and left femur and tibia. Also, increased radiotracer uptake was observed in the left clavicle. The DXA scan showed osteopenia, with a lumbar spine area BMD (Bone Mineral Density) T score -1.4 and femoral neck BMD T score -1.6.

### Mutation analysis – Ion Torrent sequencing

The leukocyte DNA analysis showed no known mutations of the examined genes, however we detected missense single nucleotide polymorphisms at exon-6 of CSF1 and exon-3 of TM7SF4 genes, which resulted in an amino acid change (Table 2).

**Table 2:** Variants with amino acid changes

Gene	Type	Ploidy	Referent	Variant	Annotation	Location	Amino acid change	SIFT score
CSF1	SNP	Het	T	C	rs1058885	EXON-6	L408P	0.17
TM7SF4^†^	SNP	Het	A	G	rs3802204	EXON-3	D349G	0.53

*SIFT – a tool that uses sequence homology to predict whether a substitution affects protein function (Sorts Intolerant From Tolerant substitutions); SNP – single nucleotid polymorphism

†also known as DCSTAMP.

The CSF1 L408P leucin to prolin amino acid change resulted in similar physico-chemical properties. Both residues were medium size and hydrophobic. The TMSF4 D349G aspartic acid to glycin amino acid change located in the cytoplasmic topological domain resulted in a change from an acidic polar amino acid to an aliphatic non-polar one. The total number of identified genetic variants in the targeted genomic region was 27 (Table 3) and based on the protein effect and allele frequency we excluded 25 variants from further investigations.

**Table 3:** List of all identified variants

Chromosome	Position	Target identifier	Type	Ploidy	Referent	Variant	Variant frequency	Coverage	dbSNP	Coding variant	Protein effect	MAF/MinorAlleleCount
chr1	110458234	CSF1	SNP	Hom	G	A	100.00	395	rs2275123	c.163-22G>A	-	A = 0.213/465
chr1	110466338	CSF1	SNP	Het	C	A	53.75	480	rs333970	c.1095C>A	p.Thr365 =	A = 0.447/977
chr1	110466466	CSF1	SNP	Het	T	C	37.40	131	rs1058885	c.1223T>C	p.Leu408Pro	C = 0.426/930
chr1	110466709	CSF1	SNP	Hom	T	C	98.13	319	rs333971	c.1466T>C	p.Phe191Ser	*t* = 0.0032/16
chr1	110466810	CSF1	SNP	Het	C	A	50.53	95	rs2229166	c.1567C>A	p.Arg225 =	A = 0.309/674
chr1	110467745	CSF1	SNP	Hom	A	G	99.84	621	rs333972	c.1623-24A>G	-	*t* = 0.0032/16
chr5	179260153	SQSTM1	SNP	Het	C	T	52.59	424	rs4935	c.624C>T	p.Asp208 =	C = 0.316/691
chr5	179260213	SQSTM1	SNP	Het	G	A	40.28	432	rs4797	c.684G>A	p.Arg228 =	G = 0.419/916
chr8	105367096	TM7SF4	SNP	Hom	T	C	99.87	1510	rs2458431	c.1030-9T>C	-	*t* = 0.400/873
chr8	105367121	TM7SF4	SNP	Het	A	G	48.78	1644	rs3802204	c.1046A>G	p.Asp349Gly	G = 0.1184/593
chr8	119941173	TNFRSF11B	SNP	Hom	A	G	99.93	1345	rs3134046	c.401-5T>C	-	A = 0.084/184
chr9	35060302	VCP	SNP	Het	T	C	52.71	848	rs684562	c.1695 + 8A>G	-	C = 0.427/932
chr9	35060955	VCP	SNP	Het	T	C	45.95	1049	rs2258240	c.1360-35A>G	-	*t* = 0.296/646
chr9	35062972	VCP	SNP	Het	C	T	46.47	2311	rs514492	c.811 + 3G>A	-	C = 0.297/649
chr9	35068201	VCP	SNP	Het	C	T	52.59	1274	rs10972300	c.129 + 47G>A	-	*t* = 0.155/339
chr10	13151224	OPTN	SNP	Het	G	A	45.83	144	rs2234968	c.102G>A	p.Thr34 =	A = 0.180/393
chr10	13152515	OPTN	SNP	Hom	T	G	98.85	611	rs79529484	c.369 + 39T>G	-	?
chr10	13158262	OPTN	SNP	Het	C	T	36.58	1282	rs2244380	c.553-5C>T	-	C = 0.205/447
chr10	13164332	OPTN	SNP	Het	T	C	57.73	1275	rs765884	c.780-53T>C	-	C = 0.191/418
chr10	13167860	OPTN	SNP	Het	G	T	49.15	411	rs676302	c.1149-86G>T	-	G = 0.199/435
chr10	13174056	OPTN	SNP	Het	T	C	24.50	547	-	c.1402-11T>C	-	-
chr13	43147671	N/A	SNP	Het	T	C	45.44	691	rs9533156	c.-149-620T>C	-	C = 0.457/999
chr13	43148024	N/A	SNP	Het	T	C	53.50	1415	rs9525641	c.-149-267T>C	-	C = 0.462/1009
chr13	43173198	N/A	SNP	Hom	T	C	99.68	937	rs3742257	c.388-1690T>C	-	*t* = 0.486/1062
chr18	60028821	TNFRSF11A	SNP	Het	G	T	20.75	877	rs35407865	c.617-92G>T	-	*t* = 0.151/330
chr18	60036083	TNFRSF11A	SNP	Hom	A	G	100.00	1187	rs8092336	c.730 + 7057A>G	-	A = 0.022/48
chr18	60051942	TNFRSF11A	SNP	Het	G	T	53.45	681	rs77857469	c.731-42G>T	-	*t* = 0.074/162

*MAF – global minor allele frequency, the MAF is actually the second most frequent allele value; SNP – single nucleotid polymorphism; hom – homozygous; het – heterozygous.

The presence of these polymorphisms has not been reported so far either in JPD or PDB. We also genotyped 5 other genes that are mostly connected with PDB (SQSTM1, TNFRSF11A, TNFRSF11B, OPTN, VCP). The seven target genes and regions were selected after a careful review of the literature on PDB (5-8). We did not detect any aberration in the examined genes and regions. Previous studies (5,6) have reported alterations only in the TNFRSF11B gene in JPD patients, independently of the phenotype of the disease. We did not detect these mutations in our patient.

### Treatment

The patient received one treatment with zoledronic acid (Novartis Pharmaceuticals Corporation, Basel, Switzerland) 5 mg intravenously. Due to vitamin D deficiency, cholecalciferol 40 000 IU/d was given for 5 days and then 2000 IU orally each day as maintenance therapy. She had a substantial response to the treatment, but did not achieve a full remission. One year after zoledronic acid treatment, the deformity in the lower limbs did not progress and no more fractures occurred. However, this period was sufficiently long for a meaningful evaluation. After zoledronic treatment, serum calcium and phosphate levels were within the reference ranges.

## Discussion

We demonstrated that NGS may help in the diagnosis of JPD. JPD, like PDB, is primarily caused by disregulation of osteoclast function. Clinically, JPD has some similarities to PDB, so it made sense to analyze the genes that had a role in the pathomechanism of PDB. Nevertheless, JPD should not be considered as a juvenile form of PDB. Increased evidence (5,6) suggests that PDB is caused by a combination of rare, high-penetrance variants in genes like SQSTM1 and TNFRSF11B, and common variants in genes such as CSF1, TNFRSF11A, and TM7SF4 (also called DCSTAMP). Genome-wide association studies (GWAS) also revealed the role of other genes, such as OPTN, VCP, and regions like rs9533156, rs9525641, rs3742257, rs3102735 in the pathogenesis of PDB (5,6). These target genes and regions were selected and analyzed in our study.

In our patient we did not detect any mutation affecting the SQSTM1 and TNFRSF11B genes, which play key roles in osteoclast differentiation and function. Former JPD case reports (9,10) have found only mutations within TNFRSF11B gene, which encodes OPG, an endogenous inhibitor of osteoclast activity (9-12). Whyte et al (13) reported 2 unrelated patients with deletion of the TNFRSF11B gene, whose serum OPG levels were undetectably low. Cundy et al described a homozygous 3-bp deletion in TNFRSF11B. Next to the 5 insertion/deletion mutations in TNFRSF11B, 3 missense TNFRSF11B mutations were identified, all being loss-of-function mutations (9). Recently, Saki et al (1) reported a kindred with TNFRSF11B mutation, which was independent of JPD phenotype.

We detected single nucleotide polymorphisms at exonal region of both CSF1 and TM7SF4 genes. The CSF1 gene encodes a macrophage colony-stimulating factor, which is essential for osteoclast and macrophage differentiation (12,14). Common genetic variants at the CSF1 gene were first identified in a GWAS as a predisposing factor for PDB (6). The mechanisms by which genetic variants of the CSF1 locus cause PDB or JPD remain unclear, but it seems likely that they act by regulating expression of CSF1, given the fact that serum M-CSF levels in affected patients are increased (2,6). A gene required for fusion of osteoclast precursors is the TM7SF4 gene. This gene encodes the dendritic cell-specific transmembrane protein (also referred as DCSTAMP), which takes part in the fusion and multi-nucleation of osteoclasts. The role of the TM7SF4 gene region in the development of PDB has been recently confirmed in an extended GWAS in PDB (6).

Our study has some limitations. Certain parts of the coding sequences of the targeted genes were not covered by the custom AmpliSeq design, thus these regions were excluded from the sequencing and variant finding. The first exon of TNFRSF11A gene was analyzed manually (primer-F: CCGCTGAGGCCGCGGCGCCC, primer-R: CTCCGCTCCCCAAAACTCCG), because 10.47% of the coding region was missed by the Ion Torrent sequencing design. We sequenced all the regions known to be linked to the Paget's disease and we did not find any of the known mutations. The effects of the found intronic variants and the functional effect of the found missense variants need to be clarified in further studies. The genetic background of JPD is still not entirely clear and missing inheritance must play a significant part in the pathogenesis of the disease. Our findings support this thesis.

In our patient we also showed a serious vitamin D deficiency (1). The low level of vitamin D also increased the bone resorbing effect, leading to the worsening symptoms. After prompt correction of the vitamin D level, while oral vitamin D3 supplementation was continued at 2000 IU/d, it was possible to treat our patient with bisphosphonate.

Several trials (6,15) reported the use of anti-resoptive drugs for treatment of JPD. However, none of these treatments was able to normalize the markers of skeletal turnover (6). One of these drugs are bisphosphonates, which appear to be safe, even if used repeatedly over a long period of time (15). Cyclical intravenous pamidronate has been reported to normalize the serum ALP level (15), while in serious cases lifelong anti-resoptive treatment may be necessary to control skeletal disease (1,16,17). Although Polyzos et al (18) described hypocalcaemia following response to zoledronate treatment in a JPD case, our patient did not have any side effect related to zoledronate treatment. After zoledronic acid treatment, her pain decreased and her quality of life also improved.

In conclusion, we showed that NGS technique can identify all the variants in several genes at the same time in a cost-effective manner. This is a new method for exploring the genetic background of juvenile Paget’s disease. We also showed that a severe vitamin D deficiency may complicate the clinical picture, so it should be treated prior to anti-resorptive therapy. Zoledronic acid was used for bisphosphonate therapy, which makes this study the second report on the use of such medication for juvenile Paget’s disease. This therapy proved to be safe and effective in this rare skeletal disease.
